# Circ_0000745 strengthens the expression of CCND1 by functioning as miR-488 sponge and interacting with HuR binding protein to facilitate the development of oral squamous cell carcinoma

**DOI:** 10.1186/s12935-021-01884-1

**Published:** 2021-05-21

**Authors:** Kuangzheng Li, Xiaosheng Fan, Ziyi Yan, Jia Zhan, Fangyun Cao, Yixia Jiang

**Affiliations:** 1 Department of Oral and Maxillofacial Surgery, The Second Affiliated Hospital, University of South China, Hengyang City, Hunan Province China; 2 Department of Respiration, The Second Affiliated Hospital, University of South China, No. 35 Jiefang Avenue, Zhengxiang District, Hengyang City, Hunan Province 421000 Peoples Republic of China

**Keywords:** Circ_0000745, OSCC, miR-488, CCND1, HuR

## Abstract

**Background:**

The implication of circular RNAs (circRNAs) in human cancers has aroused much concern. In this study, we investigated the function of circ_0000745 and its potential functional mechanisms in oral squamous cell carcinoma (OSCC) to further understand OSCC pathogenesis.

**Methods:**

The expression of circ_0000745, miR-488 and cyclin D1 (CCND1) mRNA was measured by quantitative real-time polymerase chain reaction (qPCR). Cell proliferation capacity was assessed by cell counting kit-8 (CCK-8) assay and colony formation assay. Cell cycle progression and cell apoptosis were determined by flow cytometry assay. The protein levels of CCND1, PCNA, Cleaved-caspase 3 and HuR were detected by western blot. Animal study was conducted to identify the role of circ_0000745 in vivo. The targeted relationship was verified by dual-luciferase reporter assay, pull-down assay or RNA immunoprecipitation (RIP) assay.

**Results:**

The expression of circ_0000745 was increased in OSCC tissues and cells. Circ_0000745 downregulation inhibited OSCC cell proliferation and induced cell cycle arrest and apoptosis in vitro, as well as blocked tumor growth in vivo. MiR-488 was a target of circ_0000745, and circ_0000745 downregulation suppressed OSCC development by enriching miR-488. Besides, circ_0000745 regulated CCND1 expression by targeting miR-488. In addition, circ_0000745 regulated CCND1 expression by interacting with HuR protein. CCND1 knockdown also inhibited OSCC cell proliferation and induced cell cycle arrest and apoptosis in vitro, and CCND1 overexpression recovered the inhibitory effects on OSCC cell malignant behaviors caused by circ_0000745 downregulation.

**Conclusions:**

Circ_0000745 regulated the expression of CCND1 partly by acting as miR-488 sponge and interacting with HuR protein, thus promoting the progression of OSCC.

## Introduction

Oral squamous cell carcinoma (OSCC), the most common type of oral cancer, is stated to be the sixth to eighth most common cancer worldwide [1]. It occurs anywhere in the mouth, including the tongue, upper and lower gums, palate and buccal mucosa [2]. The incidence of OSCC is on the rise globally and is the main cause of death. Despite advances in technology and treatment options, the survival rate of OSCC patients has not increased significantly (< 50% in the last three decades) [1, 3]. Smoking is the most important cancer risk factor, accounting for about 22% of cancer deaths [4]. With the progress of studies on OSCC pathogenesis, new molecular markers attract much attention, which can be used to predict the prognosis of patients and estimate the overall survival rate of different cancers. Thus, it is necessary to explore new molecular markers for the treatment of OSCC.

Recent advances have shown that circular RNAs (circRNAs) are potential readable biomarkers with multiple irreplaceable advantages. CircRNAs are widely regarded to be derived from precursor mRNAs, harboring covalently closed and single-stranded structures [5]. Accumulating evidence has identified the potential role of circRNAs in human diseases even cancers, and some circRNAs are emerged to have biological functions and clinical implications [6]. In OSCC, for example, low expression of circ_001242 was associated with tumor size and TNM stage [7]. Besides, circ_0092125 was downregulated in OSCC, and low circ_0092125 expression led to shorter overall survival in OSCC patients [8]. The involvement of circRNAs in biological processes is implicated with various mechanisms, such as RNA interaction, protein interaction, transcription or splicing regulation [5]. Commonly, circRNAs mechanically function as sponges of target microRNAs (miRNAs) or interact with RNA-binding proteins [9, 10]. By reviewing the previous studies, we found that circ_0000745 played carcinogenic effects in various cancers [11,13]. Interestingly, circ_0000745 was also mentioned to be upregulated in OSCC by circRNA microarray data [14]. However, the detailed functions of circ_0000745 in OSCC development were still lacking. Based on these, we committed to exploring the capabilities of circ_0000745 whose functions were not fully understood in OSCC.

By the advances of bioinformatics, miRNAs targeted by circRNAs can be easily obtained. Interestingly, miR-488 is predicted as a target of circ_0000745. MiR-488 was demonstrated to inhibit the migration and invasion of tongue squamous cell carcinoma (TSCC) cells [15], hinting that miR-488 might also play functions in OSCC. However, relative studies were lacking, and the interaction between circ_0000745 and miR-488 was also not confirmed.

Cyclin D1 (CCND1) is a member of the cyclin family and well-known to be essential for the transition from the G1 phase to the S phase in cell cycle [16]. CCND1 was frequently reported to be linked to tumorigenesis and aggressiveness of human cancers [17], including OSCC [18]. The underlying functional mechanism of CCND1 in OSCC remains elusive. It is well-acknowledged that miRNAs possess extensive regulatory capacities by binding to the 3 untranslated regions (3UTR) of downstream mRNAs [19]. Bioinformatics analysis exhibited that miR-488 bound to CCND1 3UTR, and their relationship needed further validation.

In this study, to determine the role of circ_0000745 in OSCC, we investigated its expression in OSCC tissues and cells and explored its function on OSCC cell growth and apoptosis *in vitro* as well as tumor growth in vivo. Moreover, we clarified that circ_0000745 regulated the expression of CCND1 by functioning as miR-488 sponge and also by interacting with RNA-binding protein (HuR). Our study further illustrated the regulatory mechanisms of circ_0000745 in OSCC.

## Materials and methods

### Tissue collection

OSCC tissues (n=64) and normal oral mucosal tissues (NC, about 2cm adjacent to cancer margin, n=64) were collected from OSCC patients who underwent surgery at The Second Affiliated Hospital, University of South China. The written informed consent was signed by each subject. All tissues were treated with liquid nitrogen and preserved at 80 storage. The study was performed with the approval of the Ethics Committee of The Second Affiliated Hospital, University of South China.

### Cell lines and cell culture

OSCC cell lines, including Cal-27, SCC-25, SCC9 and HSC-3, and human oral keratinocytes (HOK) were purchased from BeNa Culture Collection (Beijing, China). Cal-27 cells and HSC-3 cells were cultured in 90% DMEM (GIBCO, Grand Island, NY, USA) containing 10% FBS (GIBCO). SCC-25 cells were cultured in 90% EMEM (GIBCO) containing 10% FBS. SCC9 cells and HOK cells were cultured in 90% RPMI1640 (GIBCO) containing 10% FBS. These cells were maintained in a 37 incubator with 5% CO_2_.

### Cell transfection

Short hairpin RNA (shRNA) targeting circ_0000745 (sh-circ_0000745#1 and sh-circ_0000745#2) for circ_0000745 downregulation and matched negative control (sh-NC) were provided by GeneCopoeia (Guangzhou, China). For miR-488 enrichment and inhibition, miR-488 mimic (miR-488), miR-488 inhibitor (anti-miR-488) and matched negative control (miR-NC and anti-NC) were obtained from Ribobio (Guangzhou, China). Small interference RNA targeting CCND1 (si-CCND1) for CCND1 knockdown and matched negative control (si-NC) were provided by GeneCopoeia. Overexpression vector pcDNA containing CCND1 (CCND1) for CCND1 overexpression and matched control (vector) were assembled by Sangon Biotech (Shanghai, China). Cal-27 and SCC-9 cells were transfected with these oligonucleotides or vectors using Lipofectamine 3000 (Invitrogen, Carlsbad, CA, USA).

### Quantitative real-time polymerase chain reaction (qPCR)

Tissues or cells were lysed using Trizol reagent (Invitrogen) to obtain total RNA. Complementary DNA (cDNA) was synthesized from RNA using TaqMan Reverse Transcription Reagents (Invitrogen) or MicroRNA Reverse Transcription Kit (Applied Biosystems, Foster City, CA, USA). Afterwards, cDNA was amplified for qPCR using Fast SYBR Green Master Mix (Applied Biosystems) through a BioRad CFX96 system (Bio-Rad, Hercules, CA, USA). Relative expression was calculated by the 2^Ct^ method, with GAPDH or U6 as an internal reference. The primers used were listed as below: circ_0000745, F: 5-GGCCAAGGGGCCTTTACAA-3 and R: 5-GTGGCACAGACCTCTCTCTT-3; miR-488, F: 5-TGCGGCTTGAAAGGCTATT-3 and R: 5-ATGGAGCCTGGGACGAGAC-3; U6, F: 5-CTCGCTTCGGCAGCACA-3 and R: 5-AACGCTTCACGAATTTGCGT-3; GAPDH, F: 5-GCACCGTCAAGGCTGAGAAC-3 and R: 5-TGGTGAAGACGCCAGTGGA-3; CCND1, F: 5-AGCTGTGCATCTACACCGAC-3 and R: 5-GAAATCGTGCGGGGTCATTG-3.

### Cell counting kit-8 (CCK-8) assay

Cells harboring various transfections were plated into a 96-well plate (510^3^ cells in each well) and further cultured for the indicated time (24h, 48 and 72h). At the end of culture-time, a total of 10 L CCK-8 reagent (Beyotime, Shanghai, China) was pipetted into each well to incubate cells for 2h. The absorbance at 450 nm was then detected using a Multiskan Ascent (Thermo Fisher Scientific, Waltham, MA, USA), and the proliferation curves were plotted to observe the proliferative potential of cells.

### Colony formation assay

To monitor cell proliferation, cells harboring different transfections were planted into a 6-well plate (250 cells in each well) and placed into a 37 incubator containing 5% CO_2_. Cells in this condition were cultured for 14 days. Therewith, colonies were rinsed by PBS (Beyotime), fixed using paraformaldehyde and stained using 0.1% crystal violet (Beyotime). Colonies were counted and recorded.

### Flow cytometry assay

The Annexin V-FITC/PI Apoptosis Detection Kit (Elabscience, Wuhan, China) was applied for apoptosis analysis. Simply put, cells were collected at 48h post-transfection and washed with PBS. Then, a total of 110^5^ cells were used and resuspended into 500 L Annexin V binding buffer. Cell suspensions were added with 5 L Annexin V-FITC and 5 L propidium iodide (PI), mixing gently. Cells were incubated for 15min in the dark. Cell apoptosis was examined using a flow cytometer (Beckman, Miami, FL, USA).

The Cell Cycle Assay Kit (Elabscience) was applied for cell cycle analysis. Simply speaking, cells were digested with trypsin and washed with PBS. Then, a total of 510^5^ cells were suspended into PBS and transferred into 1.2 mL absolute ethyl alcohol, fixing overnight at 20 . Cells were washed with PBS, treated with RNase A and then incubated with PI solution for 30min in the dark. Cell cycle distribution was examined using a flow cytometer (Beckman).

### Western blot

Total protein was isolated using RIPA lysis buffer (Beyotime) and then separated by 12% SDS-PAGE. The protein was transferred onto PVDF membranes (Bio-Rad) and subjected to QuickBlock Blocking Buffer (Beyotime). The membranes staining protein bands were incubated with the primary antibodies, including proliferating cell nuclear antigen (PCNA) antibody (ab18197; Abcam, Cambridge, MA, USA), Cleaved-caspase 3 antibody (ab32042; Abcam), CCND1 antibody (ab226977; Abcam), GAPDH antibody (ab9485; Abcam) and HuR antibody (ab238528; Abcam). On secondary day, the membranes were incubated with goat anti-rabbit secondary antibody (ab205718; Abcam). The indicated proteins were emerged using an enhanced chemiluminescent (ECL) kit (Thermo Fisher Scientific).

### Animal studies

The Animal Care and Use Committee of The Second Affiliated Hospital, University of South China approved the animal studies. The experimental Balb/c mice (female, n=10) were purchased from Beijing HFK Bioscience Co., Ltd (Beijing, China) and housed in a pathogen-free room. Sh-circ_0000745 or sh-NC was packaged into a lentiviral vector by GeneCopoeia. Lentivirus buffer containing sh-circ_0000745 or sh-NC was used to infect Cal-27 cells. The infected Cal-27 cells (210^6^ cells in each mouse) were subcutaneously implanted into nude mice. Tumor volume (lengthwidth^2^1/2) was measured once a week. After 5 weeks, we sacrificed all mice and excised tumor tissues. Tumor tissues were weighted and used for further experiments.

### Subcellular location assay

The Cytoplasmic and Nuclear RNA Purification Kit (Norgen Biotek, Thorold, Canada) was applied to isolate RNA from cytoplasmic and nuclear fractions. The expression of circ_0000745 in different fractions was detected by qPCR. GAPDH and U6 were used as the internal inference in the cytoplasm and nucleus, respectively.

### Bioinformatics tools

Bioinformatics tools, including starbase (http://starbase.sysu.edu.cn/) [20], circBANK (http://www.circbank.cn/) [21], and circinteractome (https://circinteractome.nia.nih.gov/) [22], were used to analyze the potential targets of circ_0000745 and miR-488.

### Dualluciferase reporter assay

The partial sequence fragments of circ_0000745 or CCND1 3UTR (including wild-type and mutant-type) were cloned into PGL4 reporter vector (Promega, Madison, WI, USA), respectively. The recombinant luciferase reporter vectors, including circ_0000745-WT, circ_0000745-MUT, CCND1-3UTR-WT and CCND1-3UTR-MUT, were used for dual-luciferase reporter assay. In brief, Cal-27 and SCC9 cells were transfected with miR-488 or miR-NC and circ_0000745-WT, circ_0000745-MUT, CCND1-3UTR-WT or CCND1-3UTR-MUT and then incubated for 48h. The Dual-Luciferase reporter assay system (Promega) was applied to detect luciferase activity in cells.

### Pulldown assay

Biotinylated miR-488 (Bio-miR-488) and negative control (Bio-NC) were constructed by Ribobio. Cal-27 and SCC9 cells were transfected with Bio-miR-488 or miR-NC. Then, pull-down assay was performed using the RNA-Protein Pull-Down Kit (Thermo Fisher Scientific). The transfected cells were collected at 48h post-transfection and lysed using lysis buffer. The lysates were incubated with dynabeads streptavidin to capture compounds affinitive to Bio-miR-488. The compounds were eluted and used for qPCR.

### RNA immunoprecipitation (RIP) assay

The Imprint RNA Immunoprecipitation Kit (Sigma-Aldrich, St. Louis, MO, USA) was applied for RIP assay. Simply put, cells were subjected to RIP lysis buffer to obtain cell lysates, and cell lysates were incubated with magnetic beads conjugated with antibodies against human argonaute2 (anti-Ago2; Sigma-Aldrich) or mouse immunoglobulin G (anti-IgG; Sigma-Aldrich). The compounds containing immunoprecipitated RNAs were isolated for qPCR analysis.

### Actinomycin D treatment

Cal-27 and SCC-9 cells harboring transfection were seeded into a 96-well plate (210^3^ cells in each well) and treated with Actinomycin D (2mg/mL; Cell Signaling Technology, Danvers, MA, USA). Afterwards, cells were collected at different time (0, 3, 6, 9 and 12h) post-treatment and used for RNA isolation and subsequent qPCR analysis.

### Statistical analysis

Data collected from three independent experiments were statistically analyzed using GraphPad Prism 7 software (GraphPad Inc., La Jolla, CA, USA). The results were expressed as meanstandard deviation (SD). Paired Students *t*-test or analysis of variance (ANOVA) was used for difference analyses between two groups or among multiple groups, respectively. The overall survival of patients was assessed by Kaplan-Meier plot and log-rank test. The correlation of expression levels between two sets was analyzed by Pearson correlation coefficient. *P*<0.05 was considered statistically significant.

## Results

### Circ_0000745 was highly expressed in OSCC tissues and OSCC-related cell lines

**Fig. 1:**
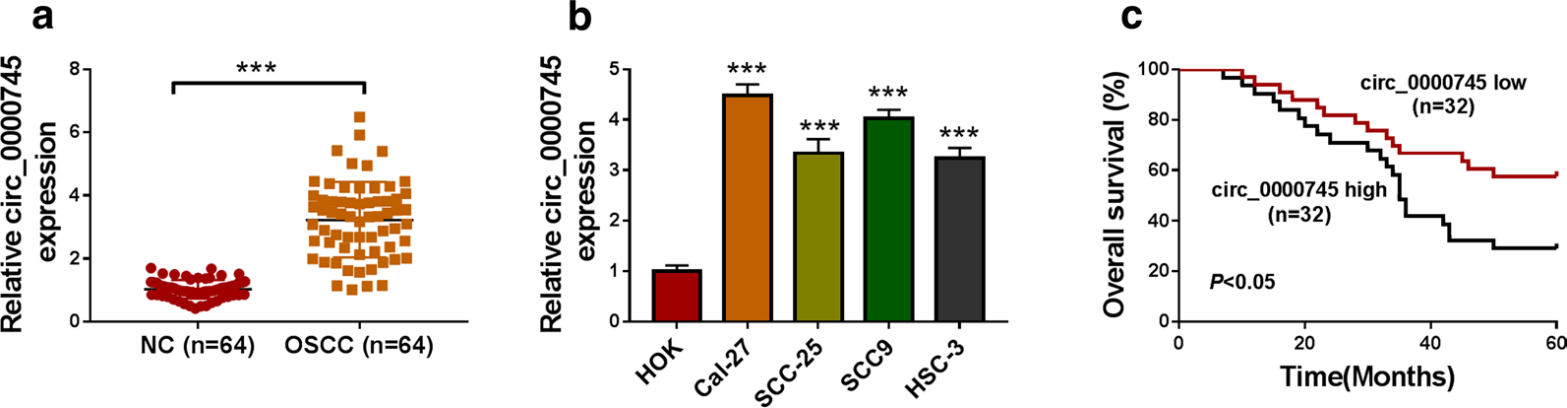
Circ_0000745 was upregulated in OSCC tissues and cells. **a** The expression of circ_0000745 in OSCC tissues (n=64) and matched normal controls (n=64) was measured by qPCR. **b** The expression of circ_0000745 in HOK, Cal-27, SCC-25, SCC9 and HSC-3 cells was measured by qPCR. **c** Overall survival was analyzed by Kaplan-Meier plot and log-rank test, using the median value of circ_0000745 expression as the cut-off value. ***P<0.001

The expression of circ_0000745 was dramatically enhanced in OSCC tissues (n=64) compared with that in normal control tissues (NC, n=64) (Fig.1a). Besides, the expression of circ_0000745 was also strikingly increased in OSCC cell lines, including Cal-27, SCC-27, SCC9 and HSC-3, compared with that in HOK cells (Fig.1b). The statistics of 5-year survival rate found that OSCC patients possessing high circ_0000745 expression harbored lower overall survival (Fig.1c). The data hinted that high circ_0000745 expression was associated with OSCC progression.

### Circ_0000745 downregulation inhibited OSCC development both in vitro and in vivo

**Fig. 2:**
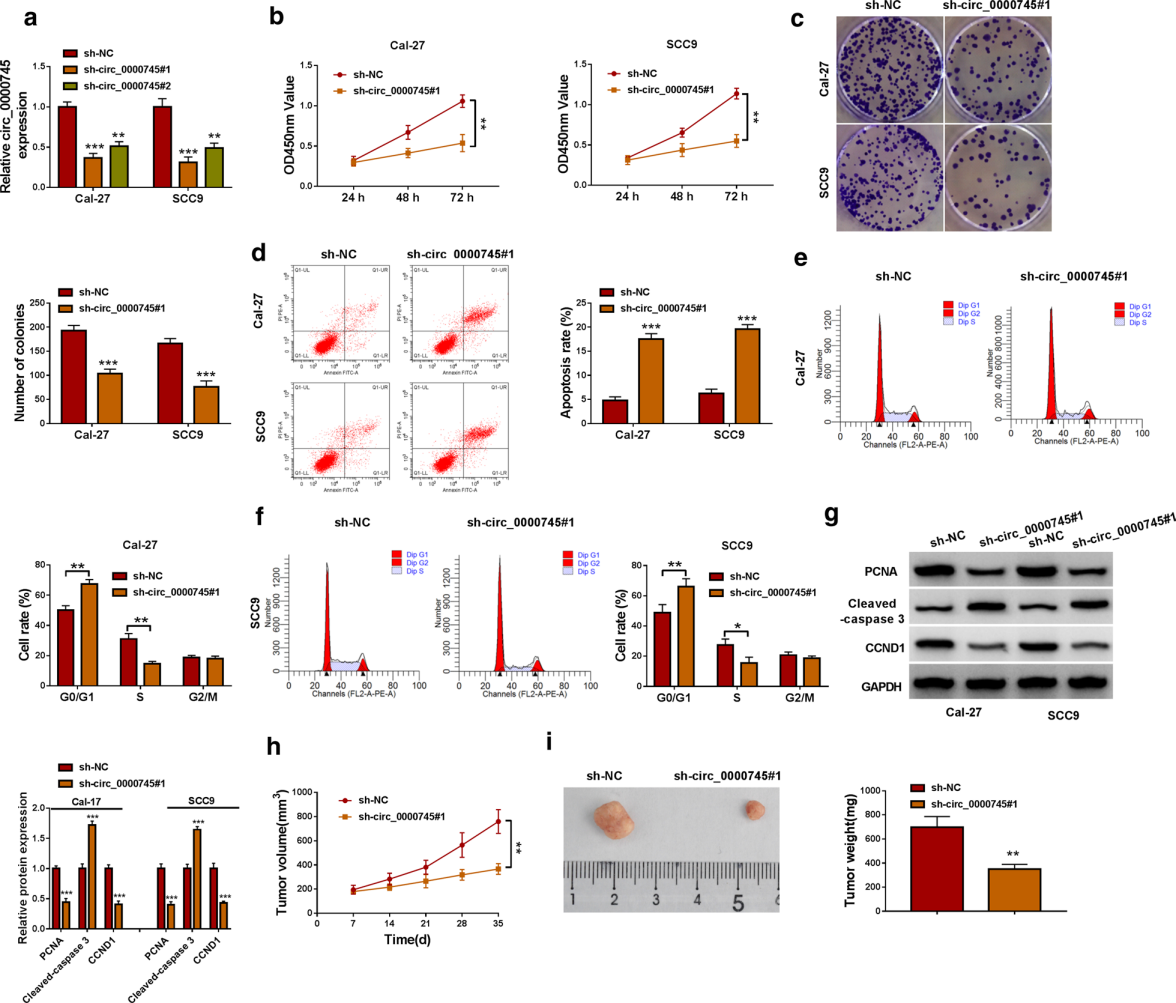
Circ_0000745 downregulation inhibited OSCC development in vitro and in vivo.**a** The efficiency of siRNA sequences targeting circ_0000745 was examined by qPCR. In Cal-27 and SCC9 cells transfected with sh-circ_0000745#1 or sh-NC, (**b**) cell proliferation was assessed by CCK-8 assay. **c** Cell proliferation was also assessed by colony formation assay. **d** Cell apoptosis was determined by flow cytometry assay. **e**,**f**Cell cycle distribution was distinguished by flow cytometry assay. **g** The protein levels of PCNA, Cleaved-caspase 3 and CCND1 were detected by western blot. **h**,** i**The role of circ_0000745 in vivo was identified by animal studies, and tumor volume and tumor weight were measured to assess tumor growth. *P<0.05, **P<0.01 and ***P<0.001

The expression level of circ_0000745 was depleted to investigate the functional role of circ_0000745. After sh-circ_0000745#1 transfection or sh-circ_0000745#2 transfection, we found that the expression of circ_0000745 was remarkably declined in Cal-27 and SCC9 cells (Fig.2a), and the role of sh-circ_0000745#1 was more significant. In CCK-8 assay, we found thatCal-27 and SCC9 cells with sh-circ_0000745#1 transfection decreased multiplication capacity (Fig.2b). Consistently, Cal-27 and SCC9 cells with sh-circ_0000745#1 transfection attenuated colony formation ability (Fig.2c). Flow cytometry assay concluded that circ_0000745 downregulation not only promoted cell apoptosis but also induced cell cycle arrest at the G0/G1 phase in Cal-27 and SCC9 cells (Fig.2d, f). Moreover, the expression levels of PCNA (a marker of proliferation) and CCND1 (a marker of cell cycle) were notably declined, while the expression level of Cleaved-caspase 3 (a marker of apoptosis) was notably enhanced in Cal-27 and SCC9 cells transfected with sh-circ_0000745#1 (Fig.2g). In animal studies, we found thatCal-27 infected with sh-circ_0000745#1 led to decreased tumor volume, tumor size and tumor weight in mice (Fig.2h, i), suggesting that circ_0000745 downregulation inhibited tumor growth. These assays suggested that circ_0000745 downregulation blocked OSCC development in vitro and in vivo.

### MiR-488 was ensured to be a target of circ_0000745 by multiple assays

**Fig. 3:**
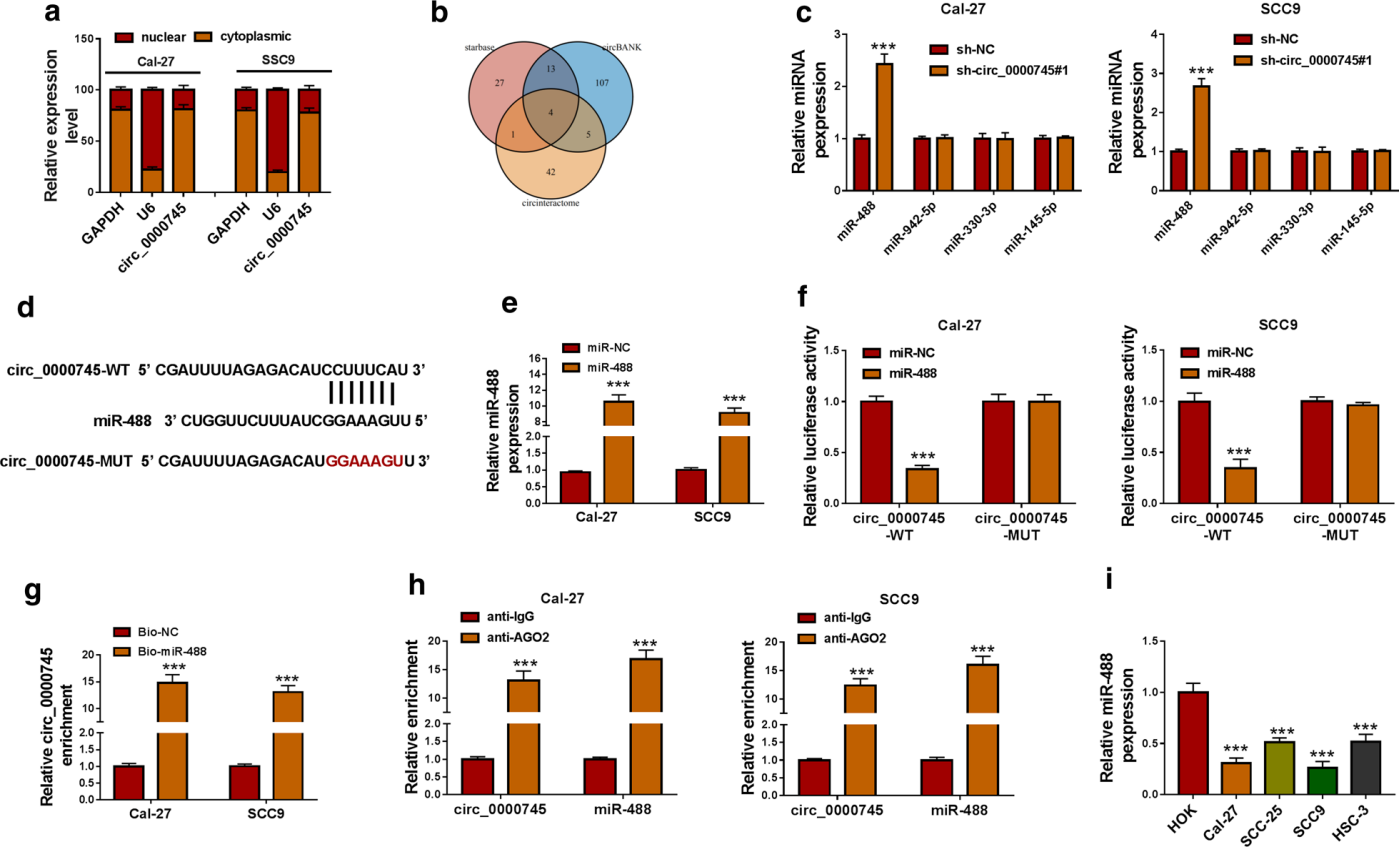
MiR-488 was screened to be a target of circ_0000745. **a** The distribution of circ_0000745 in the cytoplasm or in the nucleus was distinguished. **b** The targets of circ_0000745 were obtained from starbase, circBANK and circinteractome. **c** The expression of four potential target miRNAs in Cal-27 and SCC9 cells after circ_0000745 knockdown was detected by qPCR. **d** The binding site between circ_0000745 and miR-488 and mutant binding site. **e** The efficiency of miR-488 mimic was examined using qPCR. The relationship between circ_0000745 and miR-488 was verified by (**f**) dual-luciferase reporter assay, (**g**) pull-down assay and (**h**) RIP assay. **i** Thde expression of miR-488 in HOK, Cal-27, SCC-25, SCC9 and HSC-3 cells was measured by qPCR. ***P<0.001

We isolated cytoplasmic RNA and nuclear RNA, and the data from qPCR showed that circ_0000745 was largely distributed in the cytoplasm but not in the nucleus (Fig.3a). We speculated that cytoplasmic circ_0000745 might act as the sponge of target miRNAs. Combined the analyses of three bioinformatics tools, including starbase, circBANK and circinteractome, four miRNAs (miR-488, miR-942-5p, miR-330-3p and miR-145-5p) were predicted by these tools (Fig.3b). Next, miR-488 was screened in the following assays because miR-488 was the only one that was significantly upregulated in Cal-27 and SCC9 cells with circ_0000745 downregulation (Fig.3c). According to the predicted binding site between circ_0000745 and miR-488, the wild-type and mutant-type reporter plasmids of circ_0000745 were constructed (Fig.3d). The efficiency of miR-488 mimic was checked, and we found miR-488 expression was strikingly increased in cells with miR-488 mimic transfection (Fig.3e). Then, dual-luciferase reporter assay presented that miR-488 and circ_0000745-WT cotransfection markedly reduced luciferase activity in Cal-27 and SCC9 cells (Fig.3f). In addition, pull-down assay showed that Bio-miR-488 could pull a large amount of circ_0000745 down (Fig.3g). Moreover, RIP assay displayed that circ_0000745 and miR-488 could be enriched by anti-AGO2 rather than anti-IgG (Fig.3h). We found the expression of miR-488 was markedly declined in OSCC cell lines (Cal-27, SCC-25, SCC9 and HSC-3) compared with that in HOK cells (Fig.3i). All verifications indicated that miR-488 was a target of circ_0000745.

### MiR-488 inhibition reversed the inhibitory effects on OSCC development caused by circ_0000745 knockdown

**Fig. 4:**
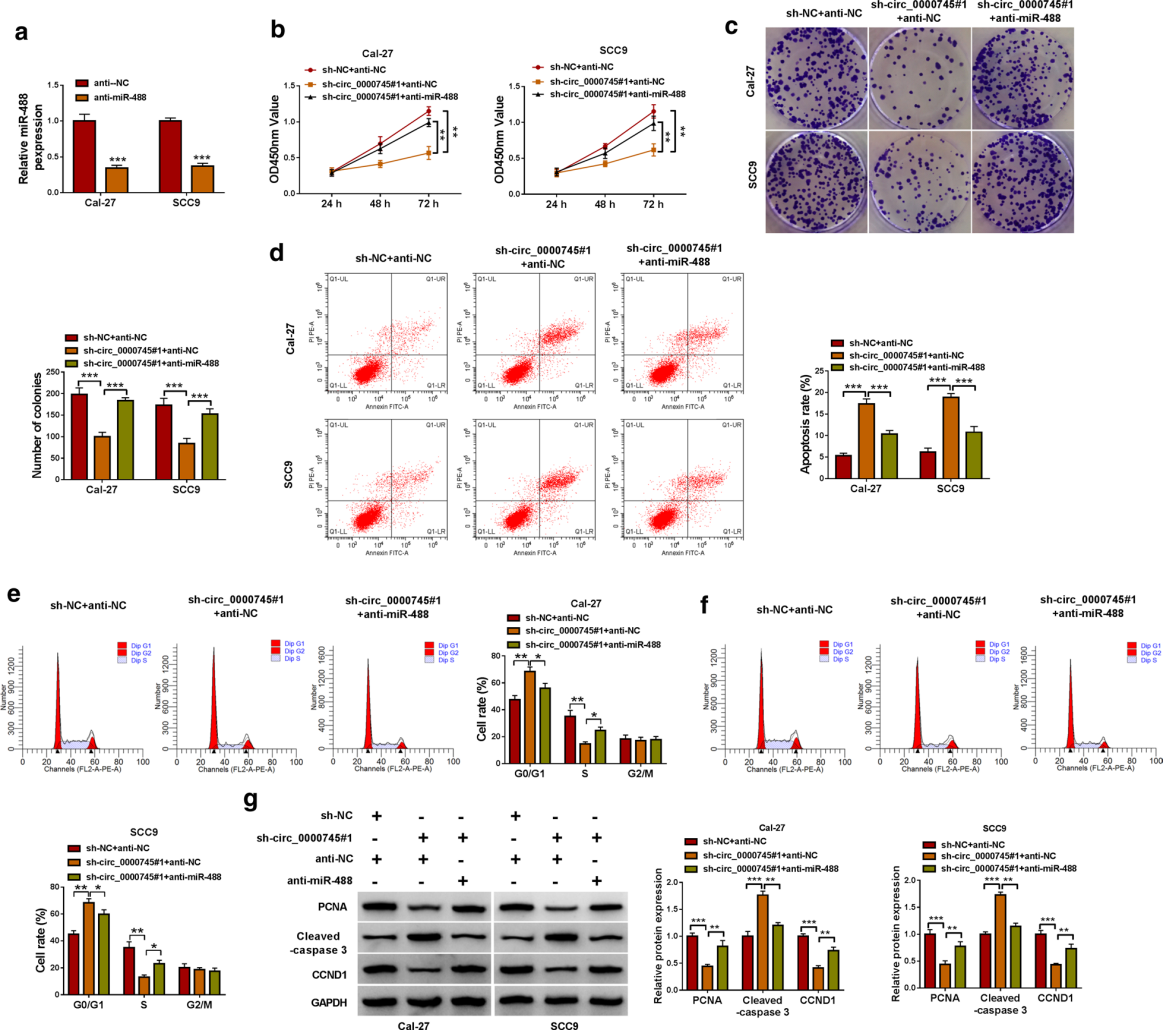
Circ_0000745 downregulation inhibited OSCC development ***in vitro*** by increasing the level of miR-488.**a** The efficiency of miR-488 inhibitor was checked by qPCR. In Cal-27 and SCC9 cells transfected with sh-circ_0000745#1+anti-NC, sh-NC+anti-NC or sh-circ_0000745#1+anti-miR-488, cell proliferation was assessed by (**b**) CCK-8 assay and (**c**) colony formation assay. **d** Cell apoptosis was identified by flow cytometry assay. **e**,**f**Cell cycle distribution at different phases was monitored by flow cytometry assay. **g** The protein levels of PCNA, Cleaved-caspase 3 and CCND1 were quantified by western blot. *P<0.05, **P<0.01 and ***P<0.001

The efficiency of miR-488 inhibitor was checked, and the expression of miR-488 was strongly decreased in Cal-27 and SCC9 cells transfected with anti-miR-488 relative to anti-NC (Fig.4a). In function, the reintroduction of anti-miR-488 substantially recovered cell proliferation capacity that was weakened by sh-circ_0000745#1 (Fig.4b). Besides, circ_0000745 downregulation-suppressed colony formation ability was restored by miR-488 inhibition (Fig.4c). Moreover, circ_0000745 downregulation-induced cell apoptosis and cell cycle arrest were largely alleviated by the additional miR-488 inhibition (Fig.4d, f). Meanwhile, the expression levels of PCNA and CCND1 were suppressed in Cal-27 and SCC9 cells transfected with sh-circ_0000745#1+anti-NC but increased in cells transfected with sh-circ_0000745#1+anti-miR-488, while the expression level of Cleaved-caspase 3 was promoted in cells transfected with sh-circ_0000745#1+anti-NC but reduced in cells transfected with sh-circ_0000745#1+anti-miR-488 (Fig.4g). All data indicated that miR-488 inhibition reversed the effects of circ_0000745 downregulation, suggesting that circ_0000745 downregulation blocked OSCC development by increasing miR-488 expression.

### CCND1 was a target of miR-488, and circ_0000745 competitively bound to miR-488 to regulate the expression of CCND1

**Fig. 5:**
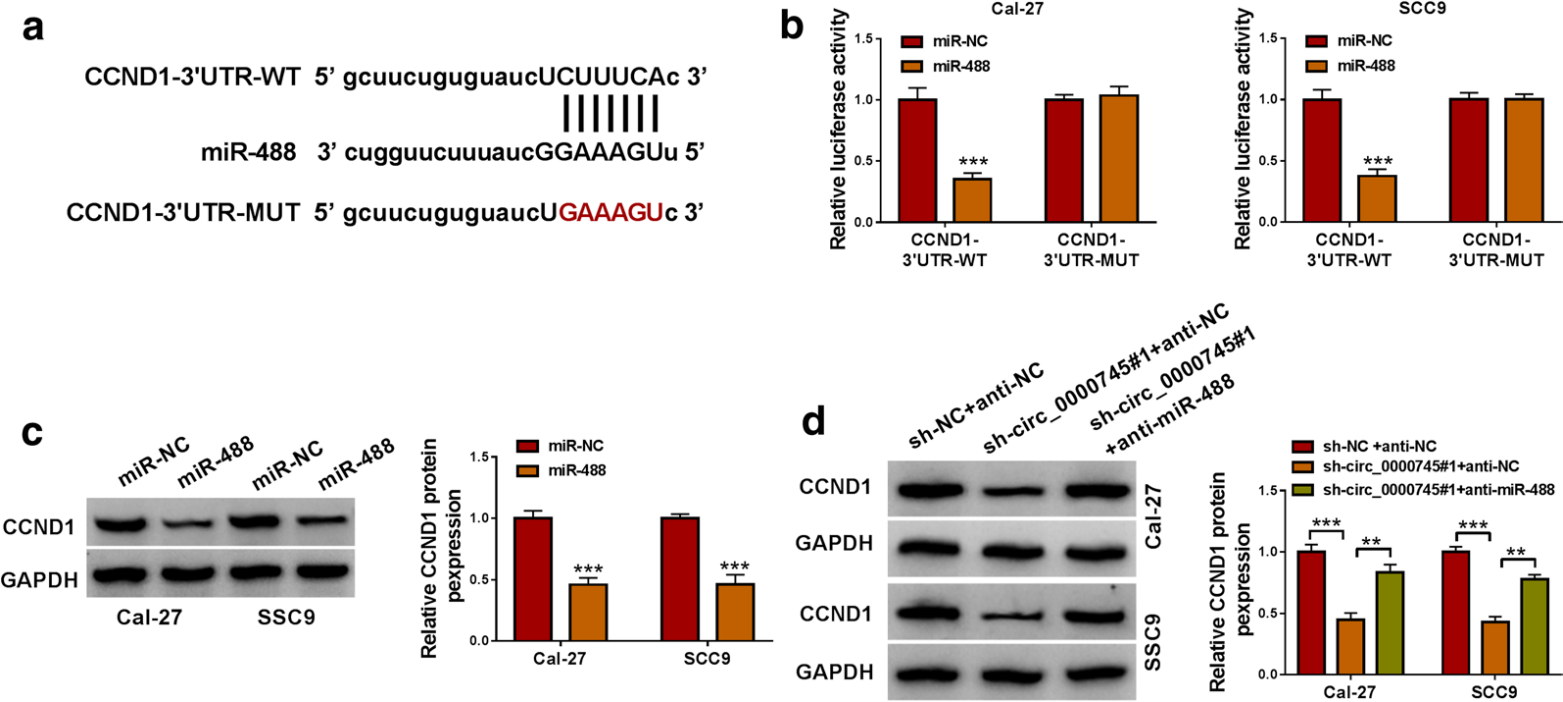
CCND1 was a target of miR-488.**a** The binding site between miR-488 and CCND1 3UTR. **b** The interaction between miR-488 and CCND1 was confirmed by dual-luciferase reporter assay. **c** The protein level of CCND1 in Cal-27 and SCC9 cells with miR-488 overexpression was detected by western blot. **d** The protein level of CCND1 in Cal-27 and SCC9 cells transfected with sh-circ_0000745#1+anti-NC, sh-NC+anti-NC or sh-circ_0000745#1+anti-miR-488 was detected by western blot. **P<0.01 and ***P<0.001

Through the prediction of starbase, we found miR-488 bound to CCND1 3UTR (Fig.5a). Dual-luciferase reporter assay showed that the cotransfection of miR-488 and CCND1-3UTR-WT could significantly decrease luciferase activity in Cal-27 and SCC9 cells (Fig.5b). Besides, miR-488 overexpression prominently impaired the expression of CCND1 protein (Fig.5c). In addition, the expression of CCND1 protein was strikingly decreased in Cal-27 and SCC9 cells transfected with sh-circ_0000745#1+anti-NC compared to sh-NC+anti-NC, while CCND1 expression was partly recovered in cells transfected with sh-circ_0000745#1+anti-miR-488 compared to sh-circ_0000745#1+anti-NC (Fig.5d). The data hinted that circ_0000745 competitively bound to miR-488 to regulate the expression of CCND1.

### Circ_0000745 interacted with HuR binding protein to regulate the expression of CCND1

**Fig. 6:**
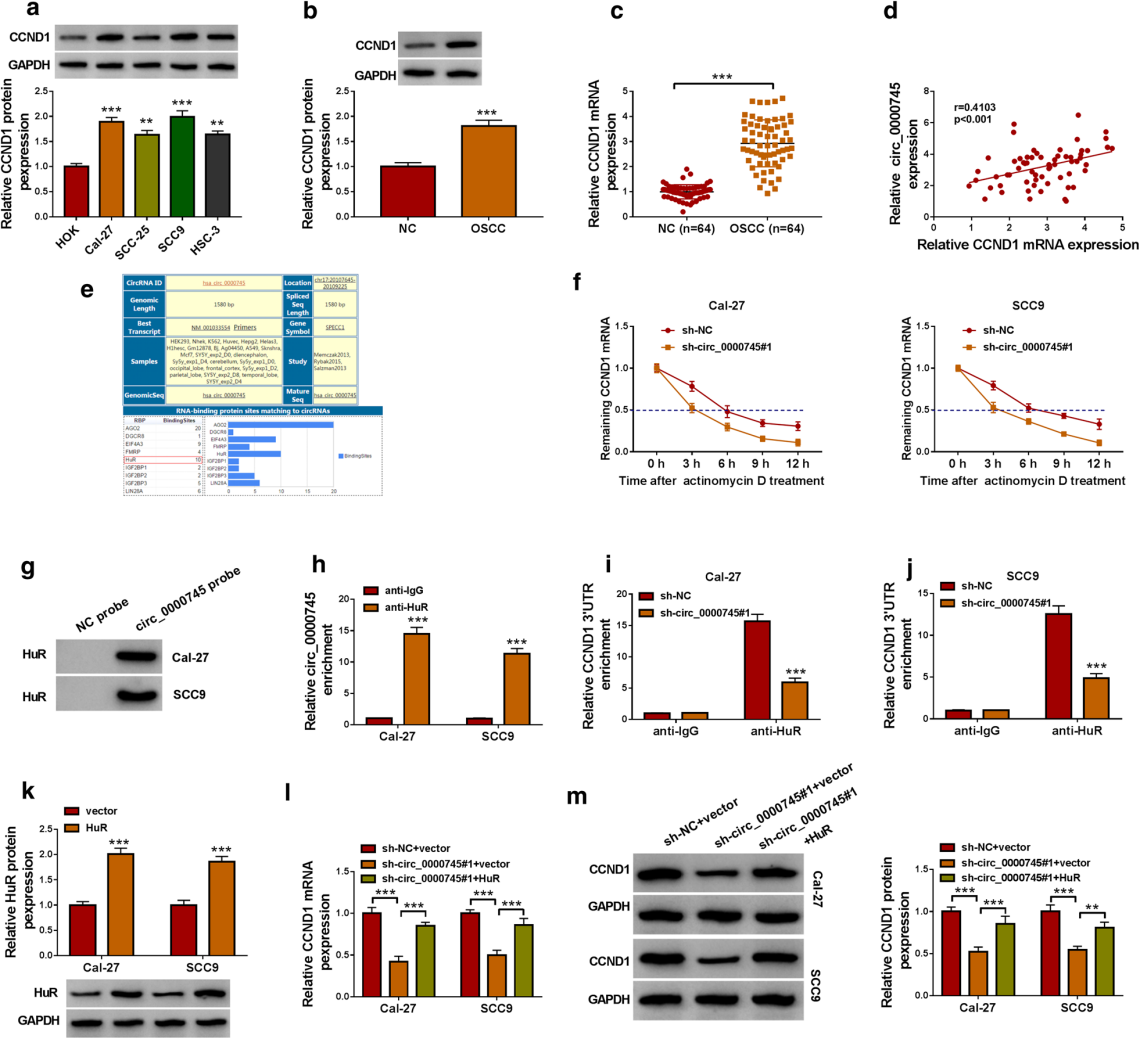
Circ_0000745 interacted with HuR to regulate the expression of CCND1.**a** The expression of CCND1 in HOK, Cal-27, SCC-25, SCC9 and HSC-3 cells was measured by western blot. **b**,**c**The expression of CCND1 in OSCC tissues and matched normal tissues was measured by western blot and qPCR. **d** The correlation between circ_0000745 expression and CCND1 expression in OSCC tissues. **e** The RNA binding proteins of circ_0000745 were provided by circinteractome. **f** The effects of circ_0000745 dysregulation on the degradation of CCND1 were assessed using actinomycin D. **g**,** h**The interaction between circ_0000745 and HuR was determined by pull-down assay and RIP assay. **i**,**j** The enrichment of CCND1 3UTR was checked in Cal-27 and SCC9 cells with sh-NC or sh-circ_0000745#1 transfection incubated with HuR or IgG antibody was detected by qPCR. **k** The efficiency of HuR overexpression was checked by western blot. **l, m** The expression of CCND1 in Cal-27 and SCC9 cells transfected with sh-NC+vector, sh-circ_0000745+vector or sh-circ_0000745+HuR was measured by qPCR and western blot. **P<0.01 and ***P<0.001

The expression of CCND1 at the protein level was pronouncedly enhanced in Cal-27, SCC-25, SCC9 and HSC-3 cells compared with that in HOK cells (Fig.6a). Besides, the expression of CCND1 was notably promoted in OSCC tissues compared with that in normal control tissues at both protein and mRNA levels (Fig.6b, c). In OSCC tissues, circ_0000745 expression showed a positive correlation with CCND1 mRNA expression (Fig.6d). The bioinformatics tool circinteractome showed that circ_0000745 could bind to HuR RNA-binding protein (Fig.6e). We guessed whether circ_0000745 regulated CCND1 expression through HuR protein. In Cal-27 and SCC9 cells transfected with sh-circ_0000745#1 or sh-NC and treated with ActD, we found sh-circ_0000745#1 transfection accelerated the degradation of CCND1 (Fig.6f). Pull-down assay showed that specific circ_0000745 probe, compared to NC probe, could effectively enrich the HuR protein (Fig.6g). The result was further verified by RIP assay (Fig.6h). Moreover, we found circ_0000745 knockdown in Cal-27 and SCC9 cells could weaken the interaction between HuR protein and CCND1 3UTR (Fig.6i, j). Then, we transfected HuR overexpression vector or empty vector into Cal-27 and SCC9 cells, and the data showed that HuR protein level was strikingly elevated after HuR transfection (Fig.6k). In addition, the expression of CCND1 at both mRNA and protein levels was markedly declined in Cal-27 and SCC9 cells transfected with sh-circ_0000745#1+vector compared with that in cells transfected with sh-NC+vector, while the expression of CCND1 was largely recovered in cells transfected with sh-circ_0000745#1+HuR (Fig.6l, m). These data suggested that circ_0000745 regulated the expression of CCND1 partly by interacting with HuR binding protein.

### CCND1 knockdown blocked OSCC development ***in vitro***

**Fig. 7:**
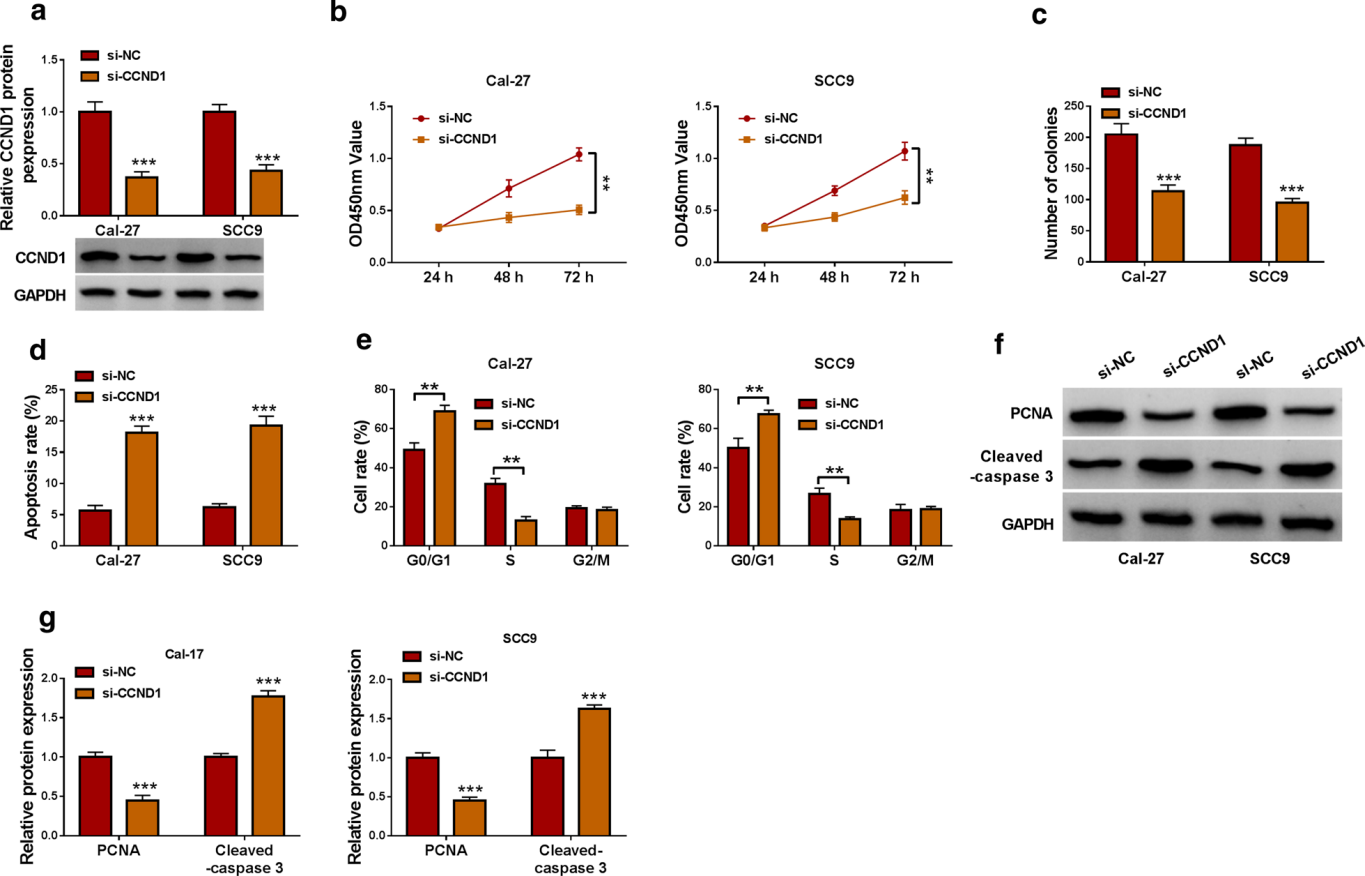
CCND1 knockdown inhibited OSCC cell growth.**a** The efficiency of CCND1 knockdown was checked by qPCR. In Cal-27 and SCC9 cells transfected with si-CCND1 or si-NC, cell proliferation was assessed by (**b**) CCK-8 assay and (**c**) colony formation assay. **d** Cell apoptosis and (**e**) cell cycle distribution were investigated by flow cytometry assay. **f**, **g**The protein levels of PCNA and Cleaved-caspase 3 in these transfected cells were quantified by western blot. **P<0.01 and ***P<0.001

The expression level of CCND1 protein was prominently declined in Cal-27 and SCC9 cells with si-CCND1 transfection (Fig.7a). Functionally, CCND1 knockdown inhibited cell proliferation and colony formation in Cal-27 and SCC9 cells (Fig.7b, c). In addition, CCND1 knockdown promoted cell apoptosis and induced cell cycle arrest at the G0/G1 phase in Cal-27 and SCC9 cells (Fig.7d, e). Moreover, CCND1 knockdown inhibited the expression of PCNA but strengthened the expression of Cleaved-caspase 3 (Fig.7f,g). All data stated that CCND1 knockdown inhibited OSCC cell growth and promoted cell apoptosis.

### Circ_0000745 knockdown weakened the expression of CCND1 to block OSCC development

**Fig. 8:**
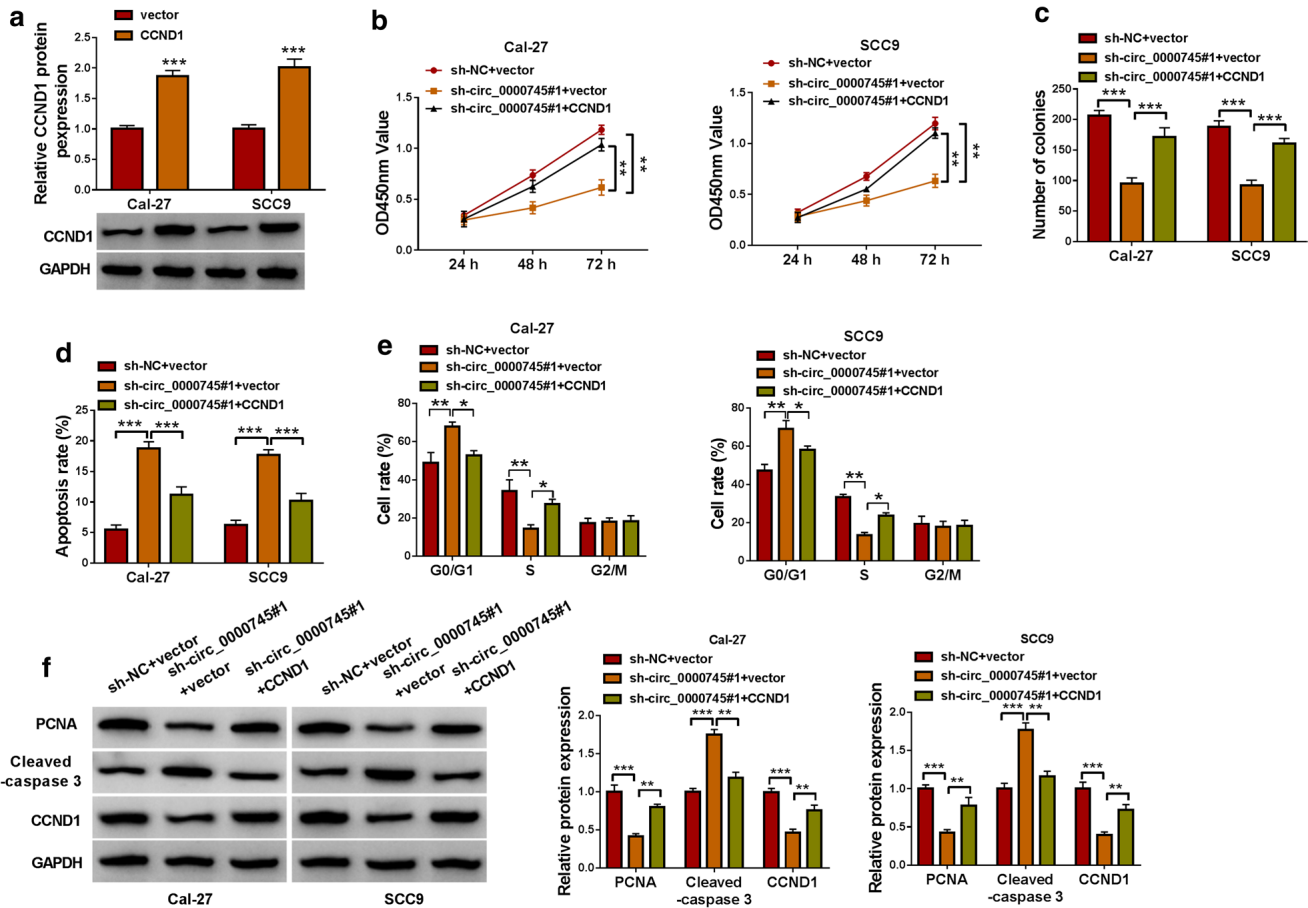
Circ_0000745 downregulation inhibited OSCC cell growth by decreasing the level of CCND1.**a** The efficiency of CCND1 overexpression was checked by western blot. In Cal-27 and SCC9 cells transfected with sh-NC+vector, sh-circ_0000745#1+vector or circ_0000745#1+CCND1, cell proliferation was monitored by (**b**) CCK-8 assay and (**c**) colony formation assay. **d**, **e**Cell apoptosis and cell cycle distribution in these cells were investigated by flow cytometry assay. **f** The protein levels of PCNA, Cleaved-caspase 3 and CCND1 in these cells were quantified by western blot. *P<0.05, **P<0.01 and ***P<0.001

CCND1 overexpression vector could notably enrich the expression of CCND1 in Cal-27 and SCC9 cells compared to the empty vector (Fig.8a). In function, CCND1 overexpression largely recovered cell proliferation capacity and colony formation ability that were blocked by circ_0000745 downregulation (Fig.8b, c). Besides, circ_0000745 downregulation-induced cell apoptosis and cell cycle arrest were notably ameliorated by the reintroduction of CCND1 rather than blank vector (Fig.8d, e). Additionally, the levels of PCNA and CCND1 were notably lessened in Cal-27 and SCC9 cells transfected with sh-circ_0000745#1+vector compared to sh-NC+vector but largely restored in cells transfected with sh-circ_0000745#1+CCND1, while the level of Cleaved-caspase 3 was notably enhanced in cells transfected with sh-circ_0000745#1+vector compared to sh-NC+vector but largely impaired in cells transfected with sh-circ_0000745#1+CCND1 (Fig.8f). These data demonstrated that circ_0000745 knockdown blocked OSCC cell malignant behaviors by degrading CCND1.

## Discussion

In order to fully understand the pathogenesis of OSCC, we explored the effects of circ_0000745 on OSCC development from the perspective of circRNA. As results, circ_0000745 expression was increased in tumor tissues and cell lines of OSCC. In function, circ_0000745 knockdown blocked OSCC development both *in vitro* and *in vivo*. Moreover, we found thatcirc_0000745 could regulate the expression of CCND1 not only by acting as miR-488 sponge but also by interacting with HuR protein, thus affecting the development of OSCC. Herein, we mainly proposed that circ_0000745 promoted the tumorigenesis and aggression of OSCC by increasing the expression of CCND1 in two ways, by decoying miR-488 or interacting with HuR.

Previous studies have illustrated the function of circ_0000745 in different cancers. For instance, circ_0000745 was highly regulated in cervical cancer, and the knockdown of circ_0000745 suppressed cervical cancer cell proliferation, migration and invasion [11, 12]. Besides, circ_0000745 expression was also elevated in leukemia cells, and forced expression of circ_0000745 induced leukemia cell proliferation and aggravated the progression of acute lymphoblastic leukemia [13]. Importantly, the abundance of circ_0000745 (circ_101996) was also shown to be increased in OSCC tissues through a circRNA microarray profile [14]. Consistent with these results, we noticed that circ_0000745 expression in OSCC tissues was higher than that in paired normal tissues, and its expression was also higher in OSCC cell lines than that in non-cancer cells. In addition, high expression of circ_0000745 was summarized to be linked to low overall survival in OSCC patients. Functional analyses revealed that circ_0000745 downregulation inhibited OSCC cell proliferation but induced OSCC cell apoptosis and cell cycle arrest *in vitro*, and circ_0000745 downregulation also impeded tumor growth *in vivo*, meaning that circ_0000745 deficiency blocked OSCC development. We concluded that circ_0000745 functioned as an oncogene to promote OSCC progression.

To address the regulatory mechanisms of circ_0000745 in OSCC, we analyzed the target miRNAs of circ_0000745. By the prediction of multiple bioinformatics softwares and the verification of various assays, we identified miR-488 as a target of circ_0000745. The data in this study presented that miR-488 was notably downregulated in OSCC tissues and cells. The inhibition of miR-488 reversed the effects of circ_0000745 downregulation and thus promoted OSCC cell growth. MiR-488 was served as a tumor suppressor, which was determined in various cancers, such as renal cell carcinoma, colorectal cancer and esophageal squamous cell carcinoma [23,25]. The evidence from this study indicated that miR-488 inhibited the development of OSCC.

Further analyses showed that miR-488 could bind to CCND1 3UTR and thus inhibited the expression of CCND1. Moreover, the expression of CCND1 was decreased in Cal-27 and SCC9 cells with circ_0000745 downregulation, while the reintroduction of miR-488 inhibitor recovered the expression of CCND1. It could be concluded that circ_0000745 regulated the expression of CCND1 by competitively targeting miR-488. CCND1 was functionally regarded as an oncogene in multiple cancers [26,28]. It has been demonstrated that CCND1 overexpression promoted OSCC cell malignant growth, mainly affecting cell cycle [29, 30]. Similarly, our data presented that CCND1 knockdown inhibited OSCC cell proliferation and promoted cell apoptosis and cycle arrest. On the contrary, CCND1 overexpression reversed the effects of circ_0000745 downregulation and thus recovered OSCC cell growth. By the analysis of RNA-binding proteins matching to circ_0000745, we found HuR possessed numerous binding sites with circ_0000745. A previous study demonstrated that circCCND1 interacted with HuR protein to elevate the expression of CCND1, thus promoting the proliferation of laryngeal squamous cell carcinoma cells [31]. Here, we verified that circ_0000745 could interact with HuR to regulate the expression of CCND1, which might be a new mechanism for circ_0000745 regulating the function of CCND1 in OSCC.

Collectively, we found that the expression of circ_0000745 was enhanced in OSCC tissues and cells, and high circ_0000745 level was associated with poor overall survivalof OSCC patients. Circ_0000745 downregulation blocked OSCC development via mediating the expression of CCND1 by acting as miR-488 sponge and inactivating with HuR protein. Our study details the function of circ_0000745 in OSCC and illustrates two mechanisms for the function of circ_0000745 in OSCC. This provides a new perspective on the pathogenesis of OSCC. However, this study is only a preliminary study of the role of circ_0000745 in OSCC and mainly discusses the functions of circ_0000745 in only two cell lines of OSCC, which is a limitation of the present study. These functional experiments should be conducted in more cell lines.

## Data Availability

Please contact the correspondence author for the data request.
